# Settling the matter of the role of vibrations in the stability of high-entropy carbides

**DOI:** 10.1038/s41467-021-25979-5

**Published:** 2021-09-30

**Authors:** Marco Esters, Corey Oses, David Hicks, Michael J. Mehl, Michal Jahnátek, Mohammad Delower Hossain, Jon-Paul Maria, Donald W. Brenner, Cormac Toher, Stefano Curtarolo

**Affiliations:** 1 Department of Mechanical Engineering and Materials Science, Duke University, Durham, NC 27708 USA; 2 Center for Autonomous Materials Design, Duke University, Durham, NC 27708 USA; 3 Department of Materials Science and Engineering, The Pennsylvania State University, University Park, PA 16802 USA; 4 Department of Materials Science and Engineering, North Carolina State University, Raleigh, NC 27695 USA

**Keywords:** Ceramics, Computational methods

## Abstract

High-entropy ceramics are attracting significant interest due to their exceptional chemical stability and physical properties. While configurational entropy descriptors have been successfully implemented to predict their formation and even to discover new materials, the contribution of vibrations to their stability has been contentious. This work unravels the issue by computationally integrating disorder parameterization, phonon modeling, and thermodynamic characterization. Three recently synthesized carbides are used as a testbed: (HfNbTaTiV)C, (HfNbTaTiW)C, and (HfNbTaTiZr)C. It is found that vibrational contributions should not be neglected when precursors or decomposition products have different nearest-neighbor environments from the high-entropy carbide.

## Abstract (web summary)

The contribution of vibrations to the stability of high-entropy ceramics is still controversial. Here the authors computationally integrate disorder parameterization, phonon modelling, and thermodynamic characterization to investigate the role of vibrations to the stability of high-entropy carbides.

## Introduction

High-entropy materials are single-phase multi-element systems with large entropy contributions, structural long-range order, and chemical disorder. They have attracted substantial interest in recent years due to their remarkable physical properties and chemical stability^1–6^. The large compositional space of high-entropy materials makes the rational computational design of disordered ceramics a challenging endeavor. From a configurational perspective, the synthesizability of high-entropy ceramics is typically assessed using ideal entropy along with the formation enthalpies from density functional theory, with simplified descriptors^7–11^, or machine learning methods^12–14^. With respect to vibrations—even if they may have a significant impact on phase stability—the contributions are drastically approximated to reduce the high computational cost, or often avoided with the hope of them being negligible, due to the technical difficulties posed in calculating them for disordered systems^15–17^. As such, in high-entropy ceramics^3^, the vibrational contributions remain a matter of conjecture—their inclusion left to researchers’ guesswork.

In the parallel field of high-entropy alloys (HEAs)^2^, a limited number of studies have produced controversial results^18–26^. Several methods have been employed, including the Debye–Grüneisen model^18,27,28^, direct phonon calculations of representative supercells^20,25,28,29^, band unfolding methods^30–33^, and machine learning^34,35^. The starting disordered configurations have commonly been chosen among Special Quasi-random Structures^36^. In these structures, the occupation correlations are optimized to resemble ideal disorder: the sampled structures aim to represent snapshots of an infinite-temperature solid solution. This situation is far from the onset of the order-disorder transition (crossing the miscibility-gap^10^), and therefore adds an extra layer of uncertainty on the driving forces stabilizing the disorder.

To capture the low-temperature behavior, Yang et al. developed the Partial Occupation (POCC) algorithm, where the representative cells can be considered snapshots of any-temperature solid-solutions^37^. The structures are statistically weighted through appropriate Boltzmann factors and symmetry degeneracies^38^ to produce ensembles determining physical properties^37^. In addition, the POCC energy spectrum can generate synthesizability descriptors, such as the entropy-forming ability (EFA). The latter was successfully used in 2018 to discover several novel five-metal high-entropy carbides (HECs) from binary precursors^9^.

This article addresses the irresolution about the role of vibrations for the stability of HECs. Here, we present a new method combining the flexibility of the POCC algorithm, full phonon characterization performed by the AFLOW Automatic Phonon Library (APL), and appropriate thermodynamic integration. As testbeds, we have chosen three disordered model carbides, $\left({{{{{{{\rm{HfNbTaTi}}}}}}}}R\right){{{{{{{\rm{C}}}}}}}}$ (*R* = V, W, Zr; HEC-*R* for short). These systems are known to form single-phase solid solutions while having large and different formation enthalpies and EFAs (Table 1). Our findings reveal that: (1) vibrations have a measurable effect on the transition temperature of HEC-W, and should thus not be neglected, but have only a small effect on HEC-V and HEC-Zr—we attribute this to the different nearest-neighbor environments in precursors and decomposition products from the high-entropy carbide; (2) the carbide sublattice distorts to accommodate the different metal cations to maximize entropy gains and minimize enthalpic penalties. The resulting force constant disorder and the mass disorder on the cation site determine the phonon properties of the metal sublattice, whereas the phonon density of states of the carbide sublattice is governed by force constant fluctuations. Although the results are related to carbides, we are confident that a similar approach can be employed to other classes of high-entropy materials, leading to practical rules about when it is necessary to include vibrational contributions and when it is acceptable to neglect them.

**Table 1:** Structural and thermodynamic parameters of the high-entropy carbide model systems.

*R*	Δ*H*_f_	EFA	VEC	*a*	mass of *R*	covalent radius	Allen electronegativity
	(meV/atom)	(meV/atom)^−1^		(Å)	(amu)	(Å)	
V	56	100	8.6	4.42 (1)	50.94	1.53	1.53
W	53	67	8.8	4.44 (2)	183.84	1.62	1.47
Zr	19	100	8.4	4.51 (8)	91.22	1.75	1.32

Energetic distance to the convex hull (Δ*H*_f_)^58^, entropy-forming-ability (EFA)^9^, valence electron concentration (VEC), and lattice parameter *a*^40^ for the chosen high-entropy carbides $\left({{{{{{{\rm{HfNbTaTi}}}}}}}}R\right){{{{{{{\rm{C}}}}}}}}$, and mass, covalent radii^43^, and Allen electronegativity^42^ of the atom *R*.

## Results

### Ensemble-averaged thermal properties

To calculate the thermal properties of chemically disordered materials, APL was integrated into AFLOW’s POCC framework^37^. The latter describes the disordered material using ordered representatives—also called derivative structures—with the desired stoichiometry using Hermite normal form matrices *U*_HNF_:

1 $${L}_{{{{{{{{\rm{POCC}}}}}}}},i}={U}_{{{{{{{{\rm{HNF}}}}}}}}}\cdot {L}_{{{{{{{{\rm{parent}}}}}}}}},$$

where *L*_parent_ and *L*_POCC,*i*_ are the unrelaxed lattices of the parent and the *i*th derivative structure, respectively. The lattices are then decorated with atoms to create the ordered representatives.

The decoration breaks the symmetry of the parent structure, causing the lattice of the derivative structure to deviate from the parent upon relaxation. The primitive lattices of the relaxed representative *L*_*i*_ can be projected onto the parent lattice using the following:

2 $${L}_{{{{{{{{\rm{parent}}}}}}}}}^{{{{{{{{\rm{proj}}}}}}}}}={\left({U}_{{{{{{{{\rm{HNF}}}}}}}}}\right)}^{-1}\cdot {L}_{i}\cdot {\left({L}_{{{{{{{{\rm{POCC}}}}}}}},i}\right)}^{-1},$$

where ${L}_{{{{{{{{\rm{parent}}}}}}}}}^{{{{{{{{\rm{proj}}}}}}}}}$ is the projected lattice.

The probabilities of the relaxed ordered representatives are determined by the Boltzmann factor:

3 $${P}_{i}\left({T}_{{{{{{{{\rm{POCC}}}}}}}}}\right)=\frac{{d}_{i}\exp \left(\frac{-{H}_{{{{{{{{\rm{f}}}}}}}},i}}{{k}_{{{{{{{{\rm{B}}}}}}}}}{T}_{{{{{{{{\rm{POCC}}}}}}}}}}\right)}{{\sum }_{j}{d}_{j}\exp \left(\frac{-{H}_{{{{{{{{\rm{f}}}}}}}},j}}{{k}_{{{{{{{{\rm{B}}}}}}}}}{T}_{{{{{{{{\rm{POCC}}}}}}}}}}\right)},$$

where *k*_B_ is the Boltzmann constant, *d*_*i*_ is the degeneracy of the *i*th derivative structure and *H*_f,*i*_ is the relative formation enthalpy *H*_f,*i*_ = *H*_f,*i*_ − *H*_f,0_ with *H*_f,0_ being the ordered representative with the lowest enthalpy. *T*_POCC_ is a virtual temperature describing the degree of disorder introduced into the system and is typically set to the synthesis temperature.

Physical properties are calculated using an ensemble average:

4 $$N({T}_{{{{{{{{\rm{POCC}}}}}}}}})=\mathop{\sum}\limits_{i}{P}_{i}({T}_{{{{{{{{\rm{POCC}}}}}}}}}){N}_{i},$$

where *N*_*i*_ is an ensemble-averageable quantity^37^.

To obtain vibrational properties such as vibrational Helmholtz free energies *F*_vib_, internal energies *U*_vib_, and entropies *S*_vib_, the phonon density of states (pDOS), *g*(*ω*), is integrated using the following equations^39^:

5 $${F}_{{{{{{{{\rm{vib}}}}}}}}}(T)={k}_{{{{{{{{\rm{B}}}}}}}}}T\int\nolimits_{0}^{\infty }{{{{{{\mathrm{log}}}}}}}\,\left(2\sinh \frac{\hslash \omega }{2{k}_{{{{{{{{\rm{B}}}}}}}}}T}\right)g(\omega )d\omega ,$$

6 $${U}_{{{{{{{{\rm{vib}}}}}}}}}(T)=\int\nolimits_{0}^{\infty }\frac{\hslash \omega }{2}\coth \left(\frac{\hslash \omega }{2{k}_{{{{{{{{\rm{B}}}}}}}}}T}\right)g(\omega )d\omega ,$$

7 $${S}_{{{{{{{{\rm{vib}}}}}}}}}(T)=\frac{{U}_{{{{{{{{\rm{vib}}}}}}}}}-{F}_{{{{{{{{\rm{vib}}}}}}}}}}{T},$$

with *ω* being the phonon frequencies. There are two possible ensemble-averageable quantities in each equation, the vibrational property itself and the pDOS *g*(*ω*). For the disordered material, there are thus two potential avenues to calculate these properties: determining them for each derivative structure and ensemble-averaging, or using the ensemble-averaged pDOS for Eqs. (5–7). Due to the linear relationship between the pDOS and *F*_vib_, *U*_vib_, and *S*_vib_, the results are independent of that choice:

$$\begin{array}{ll}{F}_{{{{{{{{\rm{vib}}}}}}}}}^{{{{{{{{\rm{avg}}}}}}}}}(T)=\mathop{\sum}\limits_{i}{P}_{i}({T}_{{{{{{{{\rm{POCC}}}}}}}}}){{F}_{{{{{{{{\rm{vib}}}}}}}}}}_{,i}\\ \qquad\qquad\qquad\quad\qquad=\mathop{\sum}\limits_{i}{P}_{i}({T}_{{{{{{{{\rm{POCC}}}}}}}}})\displaystyle\int\nolimits_{0}^{\infty }f(T,\omega ){g}_{i}(\omega )d\omega \\ \qquad\qquad\qquad\quad\qquad=\displaystyle\int\nolimits_{0}^{\infty }f(T,\omega )\mathop{\sum}\limits_{i}{P}_{i}({T}_{{{{{{{{\rm{POCC}}}}}}}}}){g}_{i}(\omega )d\omega \\ \qquad\qquad\;\;=\displaystyle\int\nolimits_{0}^{\infty }f(T,\omega ){g}^{{{{{{{{\rm{avg}}}}}}}}}(\omega )d\omega \end{array}$$

Here, ${F}_{{{{{{{{\rm{vib}}}}}}}}}^{{{{{{{{\rm{avg}}}}}}}}}$ and *g*^avg^ are the ensemble-averaged vibrational free energy and pDOS, respectively, and $f(T,\omega )={k}_{{{{{{{{\rm{B}}}}}}}}}T{{{{{{\mathrm{log}}}}}}}\,\left(2\sinh \frac{\hslash \omega }{2{k}_{{{{{{{{\rm{B}}}}}}}}}T}\right)$. The same relationship can be shown for *U*_vib_ and *S*_vib_. This work will ensemble-average the pDOS to calculate vibrational properties.

Electronic energies such as the electronic Helmholtz free energy *F*_elec_, internal energy *U*_elec_, and entropy *S*_elec_ can be calculated analogously with the ensemble-averaged electronic density of states, eDOS, *g*_e_(*ϵ*):

8 $${F}_{{{{{{{{\rm{elec}}}}}}}}}(T)={U}_{{{{{{{{\rm{elec}}}}}}}}}-T{S}_{{{{{{{{\rm{elec}}}}}}}}},$$

9 $${U}_{{{{{{{{\rm{elec}}}}}}}}}(T)=\int\nolimits_{0}^{\infty }{f}_{{{{{{{{\rm{FD}}}}}}}}}(\epsilon ,T){g}_{{{{{{{{\rm{e}}}}}}}}}(\epsilon )\epsilon d\epsilon -\int\nolimits_{0}^{{E}_{{{{{{{{\rm{F}}}}}}}}}}{g}_{{{{{{{{\rm{e}}}}}}}}}(\epsilon )\epsilon d\epsilon ,$$

10 $${S}_{{{{{{{{\rm{elec}}}}}}}}}(T)=-{k}_{{{{{{{{\rm{B}}}}}}}}}\int\nolimits_{0}^{\infty }{g}_{{{{{{{{\rm{e}}}}}}}}}(\epsilon ){s}_{T}(\epsilon )d\epsilon ,$$

where *f*_FD_(*ϵ*, *T*) is the Fermi-Dirac distribution, *E*_F_ is the Fermi energy and ${s}_{T}(\epsilon )={f}_{{{{{{{{\rm{FD}}}}}}}}}{{{{{{\mathrm{log}}}}}}}\,({f}_{{{{{{{{\rm{FD}}}}}}}}})+(1-{f}_{{{{{{{{\rm{FD}}}}}}}}}){{{{{{\mathrm{log}}}}}}}\,(1-{f}_{{{{{{{{\rm{FD}}}}}}}}})$ is the electronic entropy of *ϵ*.

These quantities, along with the configurational entropy *S*_conf_, can be used to calculate the zero-pressure Gibbs free energy, *G*:

11 $$G(T)=H-T{S}_{{{{{{{{\rm{conf}}}}}}}}}+{F}_{{{{{{{{\rm{vib}}}}}}}}}(T)+{F}_{{{{{{{{\rm{elec}}}}}}}}}(T),$$

with *H* being the enthalpy of the disordered material. For *S*_conf_, the high-temperature ideal configuration entropy ${S}_{{{{{{{{\rm{conf}}}}}}}}}=-{k}_{{{{{{{{\rm{B}}}}}}}}}{\sum }_{i}{c}_{i}{{{{{{\mathrm{log}}}}}}}\,{c}_{i}$ will be used where *c*_*i*_ is the concentration of the atom *i*.

### Structural analysis

**Fig. 1:**
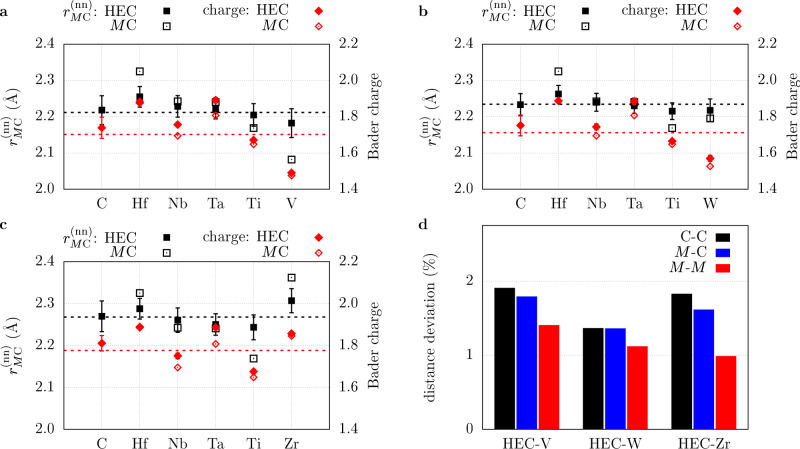
Distances as charges in high-entropy carbides. Nearest-neighbor metal-carbon distances ${r}_{M{{{{{{{\rm{C}}}}}}}}}^{({{{{{{{\rm{nn}}}}}}}})}$ (black) and magnitude of the Bader charges (red) in high-entropy carbides $\left({{{{{{{\rm{HfNbTaTi}}}}}}}}R\right){{{{{{{\rm{C}}}}}}}}$ (HEC-*R*) and their constituent rock-salt binaries *M*C: **a** HEC-V, **b** HEC-W, **c** HEC-Zr. The dashed lines represent the average between the binary carbides. The error bars represent standard deviations and are smaller than the point size for the individual metal charges. The data points for C display nearest-neighbor carbon-metal distances. **d** Relative standard deviation of the carbon−carbon (C–C), metal-carbon (*M*–C), and metal-metal (*M*–*M*) distance distributions within the HECs.

Calculating vibrational properties relies on an accurate description of the structure. It is thus crucial to evaluate whether POCC provides a reasonable structural model for the HECs. The lattice parameters of the HECs, obtained via Eq. (2), are 4.44(1) Å, 4.466(9) Å, and 4.538(5) Å for HEC-V, HEC-W, and HEC-Zr, respectively, and are in excellent agreement with experimental values (see Table 1 and ref. ^40^). The variations between the individual derivative structures represent fluctuations in interplanar spacing and manifest as peak-broadening in the X-ray diffraction patterns^9^. The average lattice lengths correlate with the charge of *R*: Fig. 1a–c shows the Bader charges of each atom (red) in the HECs^41^. While the charges of the common cations are the same between the different carbides, the charges of *R* increase slightly from V (1.49) to W (1.57) and then strongly to Zr (1.86), consistent with their Allen electronegativities^42^ and in line with the lattice parameters. They also correlate well with the average covalent radius of the metal atoms in the HECs^43^. Valence electron concentration, however, does not follow the same trend and is thus not responsible for the structural changes.

The chemical and charge disorder on the metal cation sublattice causes the carbide anion sublattice to distort, consistent with what was found in high-entropy oxides^44–46^. This can be demonstrated using the variations in interatomic distances: Fig. 1a–c shows the nearest-neighbor metal-carbon distances (black), ${r}_{M{{{{{{{\rm{C}}}}}}}}}^{({{{{{{{\rm{nn}}}}}}}})}$, in the HECs. The standard deviations for ${r}_{M{{{{{{{\rm{C}}}}}}}}}^{({{{{{{{\rm{nn}}}}}}}})}$ are generally larger for carbon than for the individual metals, indicating that the octahedral environment around the carbon atoms is more distorted than the environment around each metal. The size of the distortions is consistent with the spread of ${r}_{M{{{{{{{\rm{C}}}}}}}}}^{({{{{{{{\rm{nn}}}}}}}})}$ in the rock-salt binary carbides. It is important to note that rock-salt is not the ground state of WC, but is used as a reference phase to compare similar nearest-neighbor environments. Figure 1d shows that the origin of these disturbances lies in the carbon sublattice: the relative standard deviations of the metal-metal distance distributions inside the HECs are significantly smaller than for the carbon-carbon distances, i.e., the cations are more evenly spaced whereas the anions are removed further from their ideal positions. The sublattice-adjustment-effect can also be observed in the charge distributions: the metal charges have very small standard deviations and retain their values from the rock-salt binary carbides, or increase slightly for Nb, Ta, and W. The charge disorder on the cation sublattice is compensated by the anions through a larger charge distribution.

The structural distortions are driven by a competition between maximizing configurational entropy gains while minimizing enthalpic penalties^47^. The former can be achieved by providing coordination environments as uniform as possible. As Fig. 1a–c show, ${r}_{M{{{{{{{\rm{C}}}}}}}}}^{({{{{{{{\rm{nn}}}}}}}})}$ in the HECs of the individual metals are closer to the average ${r}_{M{{{{{{{\rm{C}}}}}}}}}^{({{{{{{{\rm{nn}}}}}}}})}$ of the binary carbides (dashed black lines), leading to more homogeneous coordination environments around the metals and thus to increased configurational entropy. On the other hand, enthalpic penalties prevent ${r}_{M{{{{{{{\rm{C}}}}}}}}}^{({{{{{{{\rm{nn}}}}}}}})}$ from being too far off their equilibrium values, leading to a distribution of nearest-neighbor distances. The competition between enthalpy and entropy is also reflected in the cation and anion charges: even though the carbide anions compensate for the charge disorder on the cation sites via a larger charge distribution, the standard deviation is still small (less than 0.1 electrons). This results in a uniform charge environment around the metal ions. To maximize configurational entropy, one would expect the cation charges to converge towards an average value (dashed red lines in Fig. 1a–c) as well. However, this would incur large enthalpic penalties. The fact that these results are corroborated by chemical and physical intuition shows that AFLOW-POCC provides a reasonable model for the structure of these HECs.

### Phonon density of states

**Fig. 2:**
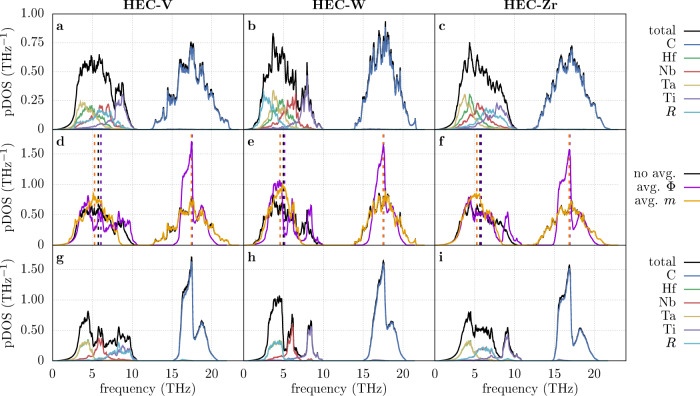
Phonon densities of states (pDOS). **a**–**c** Total pDOS for the high-entropy carbides (HECs) $\left({{{{{{{\rm{HfNbTaTi}}}}}}}}R\right){{{{{{{\rm{C}}}}}}}}$ (HEC-*R*) for *T*_POCC_ = 2473 K and their atomic projections. **d**–**f** Comparison of the total pDOS without averaging, with averaged force constants Φ, and with averaged cation masses *m*. Dashed vertical lines denote average frequencies. **g**–**i** Total and atom-projected pDOS for the HECs with averaged force constants.

The pDOS were calculated for each ordered representative and ensemble-averaged with *T*_POCC_ = 2473 K, the synthesis temperature used in the literature^9,40^. Some derivative structures for HEC-V and HEC-W show imaginary frequencies. These modes, confirmed by FROZSL calculations^48^, would make up less than 1% of the integrated averaged pDOS. The structures containing these modes were excluded from the ensemble average—while this introduces deviations in the thermodynamic properties of HEC-W, they do not change the conclusions of this article (see Supplementary Fig. 1 and the surrounding discussion). A full treatment of these structures would require including anharmonic contributions, e.g., by using self-consistent phonon theory, which is prohibitively expensive for materials as complex as these HECs. The pDOS for the HECs are shown in Fig. 2a–c. Both total and projected pDOS of the HECs are broad and largely featureless due to structural and chemical disorder—in contrast, the binary carbides show sharp features in their pDOS (see Supplementary Fig. 2a–c). Each HEC exhibits a phonon gap between the metal and carbon contributions, with the latter appearing at higher frequencies because of its low mass.

While the frequency range of the metals overall inversely correlates with their mass, the V contributions in HEC-V overlap strongly with Nb even though Nb (93 amu) has a much higher mass than V (51 amu). In addition, Zr (91 amu) in HEC-Zr traces closer with Ti (49 amu) than with Nb. In general, phonon frequencies decrease with increasing volume (larger interatomic distances) as longer bonds become softer. As Fig. 1 shows, both V and Ti have larger nearest-neighbor distances in the HECs compared to their binary counterparts whereas the distances for Zr are shortened. Using the median frequency as a rough estimate, this relationship between frequency shift and distance shift can be shown for most metals (see Supplementary Fig. 2d) and is especially strong for Zr and V.

The phonon broadening in the carbon pDOS is the consequence of force constant disorder, whereas, for the metals, it is governed by both force constant and mass fluctuations. The effect of both types of disorder on the pDOS is shown in Fig. 2d–f. Including only force constant fluctuations (average *m*, orange) narrows the metal frequency spectrum and lowers the average frequencies (dashed vertical lines), consistent with the behavior found in HEAs^31^. The carbon pDOS is unaffected since there is no mass disorder on the anion site.

To assess the impact of the force constant disorder, the interatomic force constants (IFCs) were averaged for each metal-metal, metal-carbon, and carbon-carbon interaction across derivative structures (due to different supercell sizes, averaging was only performed over representatives with the same HNF matrix). Including only mass fluctuations (average IFCs, purple) slightly elevates the average metal frequency. As the force constants become indistinguishable, the projected pDOS of the metals segregate according to their masses—shown in Fig. 2g–i—creating gap-like features in the pDOS. The total metal contributions show different degrees of broadening: the largest can be found in HEC-V and the smallest in HEC-W. This is consistent with the mass-fluctuation phonon scattering parameter, an indicator for the extent of the remaining mass disorder^49^:

12 $${{{\Gamma }}}_{m}=\mathop{\sum}\limits_{i}{c}_{i}\frac{{\left({m}_{i}-\bar{m}\right)}^{2}}{{\bar{m}}^{2}},$$

where *c*_*i*_ and *m*_*i*_ are the concentration and mass of atom *i*, respectively, and $\bar{m}$ is the average mass of the atoms on the cation sublattice. The values for Γ_*m*_ are 0.286, 0.168, and 0.198 for HEC-V, HEC-W, and HEC-Zr, respectively. There are also profound differences in the carbide region: the broad, featureless pDOS gets reduced to a width of about 5 THz with distinct maxima. The average frequency, however, does not change.

**Fig. 3:**
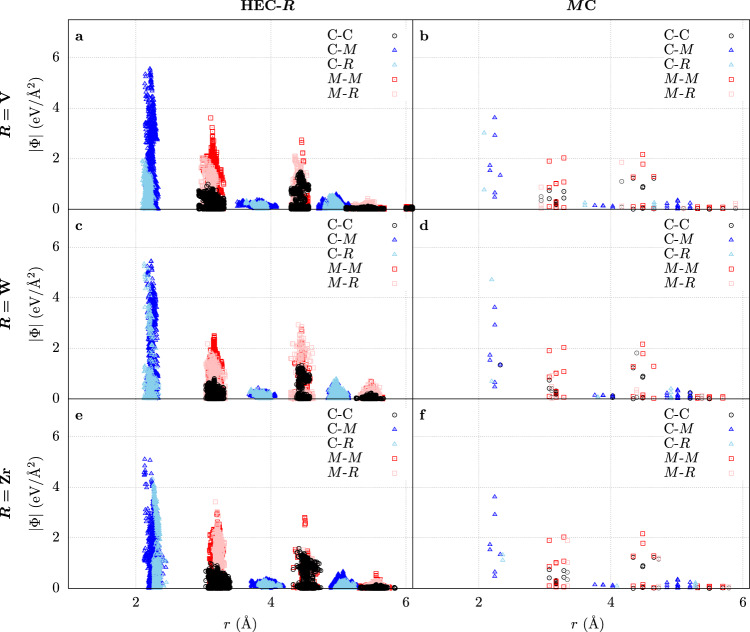
Interatomic force constants. Force constants Φ for the high-entropy carbides $\left({{{{{{{\rm{HfNbTaTi}}}}}}}}R\right){{{{{{{\rm{C}}}}}}}}$ (HEC-*R*, left) and the rock-salt binary carbides *M*C (right): **a**, **b** HEC-V, **c**, **d** HEC-W, **e**, **f** HEC-Zr. Lighter colors represent IFCs containing *R*. IFCs were projected onto common Cartesian axes using AFLOW-XtalFinder^93^.

Force constant fluctuations arise from the chemical disorder on the cation sublattice, as well as the structural distortions due to the anion sublattice accommodating the different metal species. This leads to broken local symmetries, bonds of varying lengths, and diversity of metal-metal interactions, all affecting the force field in the high-entropy material. Figure 3 shows the effect of these disturbances on the high-symmetry rock-salt structure. While the force constant patterns are similar between the HECs and the binaries, there is extensive smearing in the high-entropy material, especially in the first coordination shell. The force constants also show the origin of the frequency shifts for Zr and V: nearest-neighbor force constants of V are significantly smaller than in the binary phase, producing lower phonon frequencies, and vice versa for Zr.

### Stability of high-entropy carbides

**Fig. 4:**
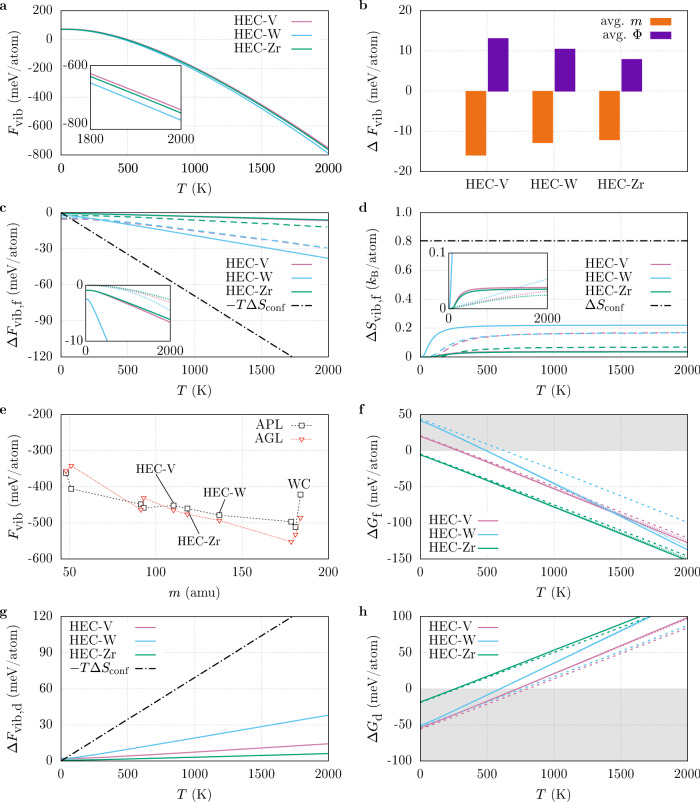
Stability of high-entropy carbides (HECs). **a** Temperature-dependent vibrational free energy *F*_vib_ of $\left({{{{{{{\rm{HfNbTaTi}}}}}}}}R\right){{{{{{{\rm{C}}}}}}}}$ (HEC-*R*) with *T*_POCC_ = 2473 K. **b** Changes in *F*_vib_ of the HECs at 1500 K when force constant disorder (avg. Φ) and mass disorder (avg. *m*) are removed. A temperature of 1500 K was chosen as entropy changes are constant in this regime (see **d**). **c** Vibrational free energy Δ*F*_vib,f_ and **d** entropy changes Δ*S*_vib,f_ for the formation reaction of the HECs from binary reactants along with configurational entropy contributions Δ*S*_conf_. Solid and dashed lines represent values obtained from the Automatic Phonon and GIBBS Libraries (APL and AGL), respectively. Insets: comparison of vibrational and electronic contributions Δ*F*_elec,f_/Δ*S*_elec,f_ (dotted lines). **e** *F*_vib_ from APL and AGL as a function of (average) cation mass *m* at 1500 K. **f** Gibbs free energy changes Δ*G*_f_ for the formation reaction of the HECs (solid lines). Δ*G*_f_ without Δ*F*_vib,f_ is shown as dashed lines. Shaded areas symbolize energies at which the HECs are unstable. **g** APL vibrational free energy changes Δ*F*_vib,d_ for the predicted decomposition reactions of the HECs compared with contributions from *S*_conf_. **h** Gibbs free energy changes Δ*G*_d_ for the decomposition reaction of the HECs (solid lines). Δ*G*_d_ without Δ*F*_vib,d_ is shown as dashed lines. Shaded areas symbolize energies at which the HECs are unstable.

The ensemble-averaged pDOS were used to calculate the vibrational free energy *F*_vib_ of the HECs as a function of temperature for *T*_POCC_ = 2473 K. The data are plotted in Fig. 4a and show that HEC-V has the highest and HEC-W the lowest *F*_vib_. In general, higher masses and larger unit cell volumes lower phonon frequencies, resulting in a more negative *F*_vib_. These mass and volume effects were also found in HEAs, even though the behavior in the investigated quinary alloys is more complex than in HEAs with fewer components^31^. With 110.2 and 118.3 amu, the average cation masses in HEC-V and HEC-Zr are very close whereas HEC-W has a higher average mass (136.8 amu). On the other hand, HEC-Zr has a significantly higher average volume (93.5 Å^3^) than HEC-V (87.3 Å^3^) and HEC-W (89.1 Å^3^). If both the mass and volume effect had an equal influence on the thermodynamic properties, HEC-W (highest mass) and HEC-Zr (largest volume) would be expected to have similar *F*_vib_. Instead, HEC-Zr traces closely with HEC-V, suggesting that the mass effect is the predominant factor for *F*_vib_ in the HECs. Another possibility is that the volume effect is compensated by the frequency shifts of V and Zr due to the expanded/contracted coordination environments.

Both mass and force constant disorder impact the thermodynamic properties as shown in Fig. 4b. *F*_vib_ is more negative when the mass disorder is removed, i.e., mass disorder destabilizes the HECs. Force constant disorder, on the other hand, has a stabilizing effect, but it is smaller compared to only including force constant fluctuations. These findings are consistent with the results found for HEAs^31^. Calculating *F*_vib_ for each sublattice separately reveals that the difference in the thermodynamic properties is exclusively caused by the metals even though force constant disorder affects both the cation and anion sublattice. This can also be demonstrated by the change in average frequencies (dashed lines in Fig. 2d–f, which shift for the metal but stay constant for the carbon contributions. The changes in vibrational properties follow no clear pattern between the HECs. Phonon frequencies can be influenced by charges and interatomic distances (via IFCs), and masses of the participating atoms. These quantities are all disordered on the cation sublattice in the HECs—an interplay of these factors is likely responsible for the observed trend.

To assess the significance of the vibrational contributions for the formation of the HECs, the difference in *F*_vib_ was calculated for the formation reaction:

$${{{{{{{\rm{HfC}}}}}}}}+{{{{{{{\rm{NbC}}}}}}}}+{{{{{{{\rm{TaC}}}}}}}}+{{{{{{{\rm{TiC}}}}}}}}+R{{{{{{{\rm{C}}}}}}}}\mathop{\longrightarrow }\limits^{{{\Delta }}{F}_{{{{{{{{\rm{vib}}}}}}}},{{{{{{{\rm{f}}}}}}}}}}({{{{{{{\rm{HfNbTaTi}}}}}}}}R){{{{{{{{\rm{C}}}}}}}}}_{5},$$

where all reactants are in their rock-salt phases, except for WC, for which the ground state hexagonal structure was used. For comparison, vibrational properties were also calculated using the Debye-Grüneisen model as implemented in the AFLOW Automatic GIBBS Library (AGL)^50^. In this approximation, *F*_vib_ is calculated as:

13 $${F}_{{{{{{{{\rm{vib}}}}}}}}}(T)=n{k}_{{{{{{{{\rm{B}}}}}}}}}T\left[\frac{9}{8}\frac{{\theta }_{{{{{{{{\rm{D}}}}}}}}}}{T}+2{{{{{{\mathrm{log}}}}}}}\,\left(1-{e}^{-\frac{{\theta }_{{{{{{{{\rm{D}}}}}}}}}}{T}}\right)-3{\left(\frac{T}{{\theta }_{{{{{{{{\rm{D}}}}}}}}}}\right)}^{3}\int\nolimits_{0}^{\frac{{\theta }_{{{{{{{{\rm{D}}}}}}}}}}{T}}\frac{{x}^{3}}{{e}^{x}-1}dx\right],$$

where *n* is the number of atoms in the cell and *θ*_D_ is the Debye temperature. The latter can be obtained via:

14 $${\theta }_{{{{{{{{\rm{D}}}}}}}}}(V)=\frac{\hslash }{{k}_{{{{{{{{\rm{B}}}}}}}}}}{\left[6{\pi }^{2}{V}^{\frac{1}{2}}n\right]}^{\frac{1}{3}}f(\nu )\sqrt{\frac{B(V)}{M}},$$

with the volume *V* and mass *M* of the unit cell. *B*(*V*) is the bulk modulus and can be obtained by isotropically expanding and compressing the unit cell and fitting the resulting DFT energies to an equation of state. *f*(*ν*) is a function of the Poisson ratio *ν* that can be calculated using AFLOW’s Automatic Elasticity Library (AEL)^50^. Figure 4c shows the difference in vibrational free energy Δ*F*_vib_ between the HECs and the binary carbides. APL (solid lines) predicts Δ*F*_vib_ to be negative for all temperatures, demonstrating that vibrations help stabilize the disordered carbides. HEC-V and HEC-Zr follow each other closely and are very small. HEC-W, on the other hand, has much stronger vibrational stabilization. The electronic contributions to the free energy (dotted lines in the inset) are smaller but of similar magnitude compared to the vibrational contributions for HEC-V and HEC-Zr, and significantly smaller than Δ*F*_vib_ for HEC-W. Except at very low temperatures, the gains in both vibrational and electronic energy are dwarfed by the energy gain due to configurational entropy changes, even for HEC-W. AGL (dashed lines) differs most from APL for HEC-V: Δ*F*_vib_ is much more negative and close to HEC-W instead of HEC-Zr.

A similar pattern emerges for the vibrational entropy change Δ*S*_vib_: the gain in vibrational entropy, shown in Fig. 4d, is small for HEC-V and HEC-Zr and levels off at 0.04 *k*_B_/atom, an order of magnitude smaller than Δ*S*_conf_. Δ*S*_vib_ in the HECs is significantly smaller than the formation and order-disorder transition entropies in HEAs^20,25,51–54^. The small changes can be explained by the atomic environments in these materials: despite the structural distortions, the first coordination shells are very similar between the binary and the high-entropy carbides, resulting in only small changes in the vibrational entropy. In HEAs, on the other hand, the chemical disorder is already found in the nearest neighbors, which contribute predominantly to the phonon properties^31^. For the same reason, Δ*S*_vib_ is much larger for HEC-W (0.22 *k*_B_/atom). In its hexagonal phase, WC has lower symmetry than its rock-salt polymorph, depressing the vibrational entropy and increasing the vibrational free energy^55^. Any rock-salt HEC with lower-symmetry precursors would experience similar stabilization, but may also incur additional enthalpy penalties because the nearest atomic environments are different than in their ground state. Other structural factors such as the mass or charge of *R* or valence electron concentration do not appear to correlate with the size of the vibrational contributions (see Table 1). The electronic entropy changes Δ*S*_elec_ (inset in Fig. 4d) nearly exceed the vibrational entropy gains for HEC-V and HEC-Zr at higher temperatures due to the small size of Δ*S*_vib_ in these systems. In HEAs, Δ*S*_elec_ is often significantly smaller (Δ*F*_elec_ is significantly less negative) than Δ*S*_vib_ (Δ*F*_vib_)^20,24,25,56^, emphasizing the impact the chemically ordered sublattice and the structural similarities with the precursors have on the vibrational thermodynamics. The aforementioned discrepancies between APL and AGL can also be found for the vibrational entropy where HEC-V shows a higher entropy gain for AGL than for APL.

The largest difference between AGL and APL can be found for HEC-V: using AGL, Δ*F*_vib_ is much more negative and close to HEC-W instead of HEC-Zr. As Fig. 4e shows, there are differences between APL and AGL when comparing the relationship between *F*_vib_ and the mass of the cation. Whereas APL shows the expected relationship of more negative *F*_vib_ with increasing mass, the relationship is not as straightforward for AGL. *F*_vib_ is more positive going from TiC (48 amu) to VC (51 amu), from ZrC (91 amu) to NbC (93 amu), and from HfC (181 amu) to TaC (184 amu). VC, NbC, and TaC show phonon anomalies with dipping dispersions near the edge of the Brillouin zone (see Supplementary Fig. 3)^57^. This anomaly is larger for VC than for NbC and TaC, consistent with the size of the drops in *F*_vib_ for APL. AGL does not probe internal modes but derives vibrational energies from mechanical properties. It does not capture these phonon anomalies, leading to more positive *F*_vib_ and smaller *S*_vib_ for these carbides. The consequence is that AGL predicts HEC-V to be more strongly stabilized by vibrations whereas Δ*F*_vib_ is small for APL. This shows that the Debye-Grüneisen model may be insufficient to accurately assess phase competitions for high-entropy materials. Figure 4f shows the impact of both vibrational and electronic contributions on the Gibbs free energy of formation Δ*G*_f_ of the HECs from the binary carbide reactants used in the synthesis of these materials^9,40^. For the entropy, the ideal high-temperature configurational entropy *S*_conf_ is used. *S*_conf_ typically overestimates the entropic contributions to Δ*G*_f_—a more precise determination of *S*_conf_ requires more expensive approaches, such as the LTVC method^10^, and is beyond the scope of this article. HEC-Zr is stable at 0 K compared to its binary precursors, whereas HEC-V and HEC-W have positive differences in enthalpy. The electronic contributions to Δ*G*_f_ are negligible and do not change the thermodynamics of the reaction. For HEC-W, on the other hand, vibrational contributions have a measurable effect: even though the configurational entropy provides the predominant contribution to the formation of HEC-W, the vibrational contributions shift the transition temperature by approximately 150 K. Vibrational contributions can thus have a significant impact on the thermodynamics of the reaction when the symmetry of the precursor is lower than the symmetry of the high-entropy material.

Using enthalpy only, AFLOW’s Convex Hull algorithm predicts that the HECs are unstable and decompose according to the following reactions^9,58^:

$$\begin{array}{lll}({{{{{{{\rm{HfNbTaTiV}}}}}}}}){{{{{{{{\rm{C}}}}}}}}}_{5}&\mathop{\longrightarrow }\limits^{{{\Delta }}{F}_{{{{{{{{\rm{vib}}}}}}}},{{{{{{{\rm{d}}}}}}}}}}&{{{{{{{{\rm{HfNbC}}}}}}}}}_{2}+{{{{{{{{\rm{TaTiC}}}}}}}}}_{2}+\frac{1}{6}{{{{{{{{\rm{V}}}}}}}}}_{6}{{{{{{{{\rm{C}}}}}}}}}_{5}+\frac{1}{6}{{{{{{{\rm{C}}}}}}}},\\ ({{{{{{{\rm{HfNbTaTW}}}}}}}}){{{{{{{{\rm{C}}}}}}}}}_{5}&\mathop{\longrightarrow }\limits^{{{\Delta }}{F}_{{{{{{{{\rm{vib}}}}}}}},{{{{{{{\rm{d}}}}}}}}}}&{{{{{{{{\rm{HfNbC}}}}}}}}}_{2}+{{{{{{{{\rm{TaTiC}}}}}}}}}_{2}+{{{{{{{\rm{WC}}}}}}}},\\ ({{{{{{{\rm{HfNbTaTiZr}}}}}}}}){{{{{{{{\rm{C}}}}}}}}}_{5}&\mathop{\longrightarrow }\limits^{{{\Delta }}{F}_{{{{{{{{\rm{vib}}}}}}}},{{{{{{{\rm{d}}}}}}}}}}&{{{{{{{{\rm{HfNbC}}}}}}}}}_{2}+{{{{{{{{\rm{TaTiC}}}}}}}}}_{2}+{{{{{{{\rm{ZrC}}}}}}}}.\end{array}$$

Figure 4g shows the change in vibrational free energy Δ*F*_vib,d_ for this reaction. Δ*F*_vib,d_ is positive for all HECs, i.e., vibrational contributions stabilize the high-entropy materials compared to their predicted decomposition products. Δ*F*_vib,d_ and Δ*F*_vib,f_ have comparable magnitudes for HEC-Zr and HEC-W because the crystal structure of HfNbC_2_ and TaTiC_2_ have similar nearest-neighbor atomic environments as in the rock-salt structure. In contrast, V_6_C_5_ has low symmetry and thus increases Δ*F*_vib,f_ for HEC-V. Vibrations alone are insufficient to stabilize this HECs: even at 2000 K, Δ*F*_vib,d_ is smaller in magnitude than the distances to the convex hull (see Table 1). Configurational entropy is a significantly larger contributor to the stability of the HECs than any change in vibrational free energy. Electronic contributions do not exceed 1 meV/atom and can thus be neglected.

The Gibbs free energies for each predicted decomposition reaction Δ*G*_d_ with and without vibrational contributions are shown in Fig. 4h. At 0 K, all HECs are unstable as Δ*G*_d_ consists only of enthalpy and zero-point energy differences. HEC-Zr is predicted to become stable near room temperature whereas the other carbides require temperatures above 600 K, emphasizing their metastability. Vibrational contributions affect the thermodynamics of the decomposition of HEC-V and HEC-W with phase transitions being shifted to lower temperatures by 80 K and 160 K, respectively. For HEC-Zr, vibrational contributions are too small to have a measurable effect.

## Discussion

To address the matter of the role of vibrations for the stability of HECs, we have combined the AFLOW Partial OCCupation algorithm with the Automatic Phonon Library. Using $\left({{{{{{{\rm{HfNbTaTi}}}}}}}}R\right){{{{{{{\rm{C}}}}}}}}$ (*R* = V, W, Zr) as test systems, our results show that: (1) The atoms in the carbide sublattice are displaced from their ideal positions to accommodate the chemical and charge disorder on the cation sublattice. This minimizes enthalpic penalties and maximizes configurational entropy gains. Phonon broadening is observed due to mass disorder on the cation sites and force constant disorder on both cation and anion sites. The disorder in the cation sublattice greatly affects the vibrational thermodynamics in these carbides: mass disorder increases the vibrational free energy whereas force constant disorder has the opposite effect. (2) Vibrations help stabilize the high-entropy materials during formation and prevent decomposition. The differences in vibrational free energy and entropy for the formation of HEC-V and HEC-Zr are found to be significantly smaller than the configurational entropy due to similar atomic environments in the high-entropy and binary materials. On the contrary, vibrational contributions in HEC-W are significant because of the lower symmetry in hexagonal WC, and they increase the temperature range in which the material is stable. The results suggest that vibrational contributions should be taken into account when one or more of the precursors or decomposition products have different nearest-neighbor environments from the high-entropy carbide. Similar rules should apply to high-entropy ceramics other than carbides. Vibrations may thus not be dismissed a priori when quantifying thermodynamic stability and miscibility in high-entropy systems.

While a change in the miscibility gap of ~150 K may not strongly affect synthesizability, it can have a significant impact on the reaction kinetics and materials properties. For example, the time-temperature-transition curve for the Fe–C system shows that the decomposition time of austenite is very sensitive to temperature and can change by several orders of magnitude within a 150 K range^59,60^. Synthesis conditions can influence mesoscopic properties such as grain sizes and thus affect the quality of the material. For instance, it was found that sintering HEC-Zr at higher temperatures leads to grain coarsening and degradation of its mechanical properties^61,62^. Moreover, the configurational entropy used is the ideal high-temperature configurational entropy, which drastically overestimates the entropy contributions at lower temperatures. We thus estimate that the shift in the transition temperature is to be understood as a lower limit, i.e., vibrations are likely to be more important if the entropy were calculated accurately.

It is important to note that these calculations use the harmonic approximation to calculate the vibrational free energy, which relies on calculations performed at 0 K. Anharmonic effects change the phonon frequencies of material and become increasingly significant at higher temperatures. They may also be able to stabilize the derivative structures with imaginary frequencies^63^, which would increase the accuracy of the ensemble averages. Including anharmonicity is computationally expensive even for simple systems, and the computational cost is prohibitive for systems with the complexity of five-metal HECs. If simple solid solutions can be a guide for high-entropy ceramics, anharmonic contributions are expected to further stabilize the disordered phase^64^, indicating again that vibrations can play a key role in the stability of high-entropy ceramics. In addition, the calculations were performed using the Perdew-Burke-Ernzerhof (PBE) functional. The choice of functional can affect the calculated phonon densities of states and thus the resulting thermodynamic properties of the material. There are multiple examples of increasing phonon frequencies and higher melting temperatures with the Strongly Constrained and Appropriately Normed Semilocal Density Functional (SCAN) compared to PBE^65–67^. However, since this work calculates mixing free energies and the predicted transition temperature is far below the melting points of these carbides, the errors in using the PBE functional may cancel out. More research is needed to investigate the effect of the functional on the transition temperatures.

The present calculations have been performed on structures representing stoichiometric samples. Experiments on HECs with variable carbon content, however, have shown that vacancies and interstitial carbon atoms affect their mechanical and thermal transport properties^68,69^. While phonons can contribute to the formation of vacancies through anharmonic effects, their energetic contribution to the stability of the material is expected to be small, especially at low temperatures^70^. In ZrC, ab-initio calculations have shown a positive Gibbs vacancy formation energy up to the melting point^71^, and it can be expected to be similar in other transition metal carbides and HECs. Nevertheless, even these small contributions can have sizeable effects on thermophysical properties such as thermal expansion and heat capacities^70,71^. Defects are thus an exciting avenue for future research that should be explored when designing new high-entropy ceramics with desired properties.

## Methods

### The AFLOW automatic phonon library

In the harmonic approximation, phonon modes are calculated by diagonalizing the dynamical matrix $D\left({{{{{{{\bf{q}}}}}}}}\right)$:

15 $$D\left({{{{{{{\bf{q}}}}}}}}\right){{{{{{{{\bf{e}}}}}}}}}_{\lambda }={\omega }_{\lambda }^{2}{{{{{{{{\bf{e}}}}}}}}}_{\lambda },$$

where the phonon mode $\lambda =\left\{{{{{{{{\bf{q}}}}}}}},j\right\}$ is a combined index consisting of the reciprocal space point **q** and the branch index *j*. *ω*_*λ*_ and **e**_*λ*_ are the frequency and the eigenvector, respectively, of the mode. The components of the dynamical matrix are^72^:

16 $${D}_{\alpha \beta }\left(\kappa {\kappa }^{\prime}| {{{{{{{\bf{q}}}}}}}}\right)=\frac{1}{\sqrt{{m}_{\kappa }{m}_{{\kappa }^{\prime}}}}\mathop{\sum}\limits_{{l}^{\prime}}{{{\Phi }}}_{\alpha \beta }\left(l\kappa ;{l}^{\prime}{\kappa }^{\prime}\right)\exp \left[i{{{{{{{\bf{q}}}}}}}}\cdot \left({{{{{{{{\bf{R}}}}}}}}}_{{l}^{\prime}}-{{{{{{{{\bf{R}}}}}}}}}_{l}\right)\right],$$

with the Cartesian indices *α* and *β*, the atomic indices *κ* and ${\kappa }^{\prime}$, and the supercell indices *l* and ${l}^{\prime}$. *m*_*κ*_ is the mass of atom *κ* and **R**_*l*_ is the vector connecting the origin of the crystal to the origin of supercell *l*. ${{{\Phi }}}_{\alpha \beta }\left(l\kappa ;{l}^{\prime}{\kappa }^{\prime}\right)$ are the harmonic IFCs:

17 $${{{\Phi }}}_{\alpha \beta }\left(l\kappa ;{l}^{\prime}{\kappa }^{\prime}\right)=\frac{{\partial }^{2}V}{\partial {{{{{{{\bf{u}}}}}}}}{\left(l\kappa \right)}_{\alpha }\partial {{{{{{{\bf{u}}}}}}}}{\left({l}^{\prime}{\kappa }^{\prime}\right)}_{\beta }},$$

where *V* is the potential energy of the crystal and ${{{{{{{\bf{u}}}}}}}}{\left(l\kappa \right)}_{\alpha }$ and ${{{{{{{\bf{u}}}}}}}}{\left({l}^{\prime}{\kappa }^{\prime}\right)}_{\beta }$ are atomic displacements. Determining these IFCs is the central problem for phonon calculations.

AFLOW calculates phonon properties with the APL module. IFCs are obtained either from Γ-point density functional perturbation theory or through the finite displacement method.

The finite displacement method applies small distortions to the atomic positions inside a supercell and calculates the forces using density functional theory (DFT). The IFCs are then calculated using central differences:

18 $$\begin{array}{lll}{{{\Phi }}}_{\alpha \beta }\left(l\kappa ;{l}^{\prime}{\kappa }^{\prime}\right) 	=\,\displaystyle\frac{{\partial }^{2}{{\Phi }}}{\partial {{{{{{{\bf{u}}}}}}}}{\left(l\kappa \right)}_{\alpha }\partial {{{{{{{\bf{u}}}}}}}}{\left({l}^{\prime}{\kappa }^{\prime}\right)}_{\beta }} \hfill\\ 	=\,\displaystyle\frac{\displaystyle\frac{\partial {{\Phi }}(+{{\Delta }}{{{{{{{\bf{u}}}}}}}}{\left(l\kappa \right)}_{\alpha })}{\partial {{{{{{{\bf{u}}}}}}}}{\left({l}^{\prime}{\kappa }^{\prime}\right)}_{\beta }}-\frac{{{\Phi }}(-{{\Delta }}{{{{{{{\bf{u}}}}}}}}{\left(l\kappa \right)}_{\alpha })}{\partial {{{{{{{\bf{u}}}}}}}}{\left({l}^{\prime}{\kappa }^{\prime}\right)}_{\beta }}}{2{{\Delta }}u}\hfill\\ 	=-\displaystyle\frac{{{{{{{{{\bf{F}}}}}}}}}_{\beta }\left({l}^{\prime}{\kappa }^{\prime},+{{{{{{{\bf{u}}}}}}}}{\left(l\kappa \right)}_{\alpha }\right)-{{{{{{{{\bf{F}}}}}}}}}_{\beta }\left({l}^{\prime}{\kappa }^{\prime},-{{{{{{{\bf{u}}}}}}}}{\left(l\kappa \right)}_{\alpha }\right)}{2{{\Delta }}u},\end{array}$$

where ${{{{{{{\bf{F}}}}}}}}\left({l}^{\prime}{\kappa }^{\prime},+{{{{{{{\bf{u}}}}}}}}{\left(l\kappa \right)}_{\alpha }\right)$ is the force on atom ${l}^{\prime}{\kappa }^{\prime}$ when atom *lκ* is displaced along *α* with length Δ*u*.

For a full set of IFCs, each symmetrically inequivalent atom needs to be displaced along with three linearly independent directions. To minimize the number of calculations, APL uses the following algorithm:

- Create test displacements along (i) the unit cell axes, (ii) the face diagonals, and (iii) the body diagonal.
- Using the site point group of the atom^38^ and Gram-Schmidt orthogonalization, create a set of displacement vectors that are orthogonal and symmetrically equivalent to the test displacement.
- Sort these sets of displacements by the number of equivalent vectors from highest to lowest.
- Take the displacements inside the first set of this sorted list. If there are less than three, add the displacements in the next set of the list and use the Gram–Schmidt method and the site point groups to create orthogonal vectors. Repeat until three linearly independent directions are found.

This method not only reduces computational requirements by minimizing the number of calculations: using site point groups to generate the displacements also leads to supercells with the highest possible symmetry.

Due to the finite nature of the DFT calculations, the force constants do not generally fulfill the acoustic sum rule:

19 $$\mathop{\sum}\limits_{{\kappa }^{\prime}}{{{\Phi }}}_{\alpha \beta }\left(l\kappa ;{l}^{\prime}{\kappa }^{\prime}\right)=0.$$

APL enforces the sum rule using the following equations:

20 $${{{\Phi }}}_{\alpha \beta }\left(l\kappa ;{l}^{\prime}{\kappa }^{\prime}\right)=\frac{1}{2}\left({{{\Phi }}}_{\alpha \beta }^{\prime}\left(l\kappa ;{l}^{\prime}{\kappa }^{\prime}\right)+{{{\Phi }}}_{\beta \alpha }^{\prime}\left({l}^{\prime}{\kappa }^{\prime};l\kappa \right)\right),$$

21 $${{{\Phi }}}_{\alpha \beta }\left(l\kappa ;l\kappa \right)=-\mathop{\sum}\limits_{\kappa \ne {\kappa }^{\prime}}{{{\Phi }}}_{\alpha \beta }^{\prime}\left(l\kappa ;{l}^{\prime}{\kappa }^{\prime}\right),$$

where ${{{\Phi }}}_{\alpha \beta }^{\prime}\left(l\kappa ;{l}^{\prime}{\kappa }^{\prime}\right)$ is the raw IFC obtained from DFT forces. Equation (20) enforces transposition symmetry, i.e., that the force constants be invariant upon exchanging (*α*, l, *κ*) and (*β*, ${l}^{\prime}$, ${\kappa }^{\prime}$), and Eq. (21) is a self-interaction term. In addition, the IFCs are symmetrized with respect to the site point group operations of the crystal:

22 $${{\Phi }}\left(\kappa ;{\kappa }^{\prime}\right)=\frac{1}{\left|G\right|}\mathop{\sum}\limits_{U}{U}^{-1}{{\Phi }}\left({l}^{\prime}{\kappa }^{\prime};{l}^{^{\prime\prime} }{\kappa }^{^{\prime\prime} }\right)U,$$

where *U* is a 3 × 3 unitary matrix describing the point group operation transforming ${l}^{\prime}{\kappa }^{\prime}$ into *l*^*″*^*κ*^*″*^ and $\left|G\right|$ is the cardinality of the point group *G*. The symmetrized force constants are then used to construct the dynamical matrix and solve Eq. (16) to obtain phonon dispersions and densities of states.

### DFT calculations

DFT calculations were performed using the AFLOW framework^73–79^ and the Vienna ab-initio simulation package (VASP)^80^. Exchange and correlation were treated with the projector augmented wave (PAW) method^81^ in the generalized gradient approximation (GGA) proposed by Perdew, Burke, and Ernzerhof (PBE)^82^. The cut-off energies were chosen to be 40% higher than the recommended maximum cut-off (ENMAX) of all constituent elements.

Phonon frequencies and eigenvectors were calculated using APL via the finite-displacement method with a displacement magnitude of 0.015 Å. Supercells were created containing at least 180 atoms. Convergence for the static calculations was achieved when the energy difference between two electronic steps was below 10^−8^ eV. To reduce the residual forces on the atoms in the supercell, structures were allowed to relax until the forces on each atom were below 10^−3^ eV/Å. Since the compounds are metallic, no polar corrections were employed. Phonon densities of states were calculated using the tetrahedron method on a 21 × 21 × 21 **q**-point grid, which was sufficient to converge vibrational properties^83,84^.

Phonon properties for the binary carbides HfC, NbC, TaC, TiC, VC, WC, and ZrC (space group: $Fm\bar{3}m$, #225; Pearson symbol: cF8; AFLOW prototype^85–87^: AB_cF8_225_a_b^88^) were calculated using a 5 × 5 × 5 supercell (250 atoms). Dispersions for WC were also calculated using its experimental structure ($P\bar{6}m2$, #187; hP2; AB_hP2_187_d_a^89^) where a 5 × 5 × 4 supercell (200 atoms) was employed. Static calculations were performed using 7000 **k**-points per reciprocal atom (KPPRA). The *k*-point density was chosen because the vibrational free energy at 300 K was converged within 1 meV/atom for all binary carbides. The phonon dispersions can be found in Supplementary Fig. 3. For the decomposition products, pDOS were calculated using KPPRA = 7000. The supercell sizes were 5 × 5 × 2 (200 atoms) for C (graphite; *P*6_3_/*mmc*, #194; hP4; A_hP4_194_bc^90^), 3 × 3 × 1 (297 atoms) for VC_0.83_ (*P*3_1_12, #151; hP33; A5B6_hP33_151_3a2b_3c), 4 × 4 × 3 (192 atoms) for TaTiC_2_ (*P*4/*mmm*, #123; tP4; A2BC_tP4_123_ad_b_c), 4 × 4 × 1 (192 atoms) for HfNbC_2_ ($R\bar{3}m$, #166; hR4; A2BC_hR4_166_c_b_a), respectively.

For $\left({{{{{{{\rm{HfNbTaTi}}}}}}}}R\right){{{{{{{\rm{C}}}}}}}}$ (*R* = V, W, Zr), derivative structures were created using the AFLOW-POCC algorithm from a rock-salt parent structure (space group: $Fm\bar{3}m$, #225; Pearson symbol: cF8; AFLOW prototype^85–87^: AB_cF8_225_a_b^88^), producing 49 ordered representative structures^9^. To determine an appropriate *k*-point grid for each, the KPPRA value for APL calculations of representative structures was increased until the vibrational free energy at 300 K was converged within 1 meV/atom. APL supercells were generated using standard conventional representations to achieve a more spherical supercell. This allows for including more full coordination shells while keeping cell sizes as small as possible. For the derivative structures, it was critical that the relaxation calculations and the static calculations to obtain the forces were done with the exact same *k*-point densities. Doing otherwise leads to an increase in residual forces by up to two orders of magnitude. This was observed in the unit cell as well, even at KPPRA values above 20,000. A summary of all calculation parameters is shown in Table 2.

**Table 2:** DFT parameters for the APL calculations of the POCC derivative structures.

POCC structure(s)	Degeneracy *g*_*i*_	Lattice type	Supercell	number of atoms	*k*-point grid supercell	*k*-point grid relaxations
01–12	10	rhl	3 × 3 × 1	270	4 × 4 × 1	12 × 12 × 1
13–24	10	orci	4 × 3 × 1	240	3 × 3 × 2	12 × 9 × 2
25–36	10	mclc	3 × 1 × 2	120	4 × 4 × 3	12 × 4 × 6
37–48	10	bct	3 × 3 × 1	180	4 × 4 × 2	12 × 12 × 2
49	120	mclc	2 × 3 × 2	240	3 × 3 × 3	6 × 9 × 6

*rhl* rhombohedral, *orci* body-centered orthorhombic, *bct* body-centered tetragonal, *mclc* base-centered monoclinic.

## Supplementary information

Supplementary Information

## Data Availability

All ab-initio data are freely available as part of the AFLOW online repository and can be accessed through the AFLOW.org REST-API Interface^91^ and AFLUX search language^92^. The code is available via the AFLOW software suite.
