# Supplementary material for: Settling the matter of the role of vibrations in the stability of high-entropy carbides
Source: Nat Commun. 2021 Sep 30;12:5747. doi: 10.1038/s41467-021-25979-5 (PMC8484556; doi:10.1038/s41467-021-25979-5)
Supplement: Supplementary file 1 — Supplementary Information [file 41467_2021_25979_MOESM1_ESM.pdf]

# Supplementary Information
# Settling the matter of the role of vibrations in the stability of high-entropy carbides

Marco Esters,[1,2] Corey Oses,[1,2] David Hicks,[1,2] Michael J. Mehl,[1,2] Michal Jahnátek,[1] Mohammad Delower Hossain,[3] Jon-Paul Maria,[3] Donald W. Brenner,[4] Cormac Toher,[1,2] and Stefano Curtarolo[1,2,*]

[1]*Department of Mechanical Engineering and Materials Science, Duke University, Durham, North Carolina 27708, USA*

[2]*Center for Autonomous Materials Design, Duke University, Durham, North Carolina 27708, USA*

[3]*Department of Materials Science and Engineering, The Pennsylvania State University, University Park, PA 16802, USA*

[4]*Department of Materials Science and Engineering, North Carolina State University, Raleigh, NC 27695, USA*

(Dated: September 3, 2021)

## SUPPLEMENTARY NOTE 1: DERIVATIVE STRUCTURES WITH IMAGINARY FREQUENCIES

For (HfNbTaTiV)C (HEC-V) and (HfNbTaTiW)C (HEC-W), some derivative structures have imaginary frequencies in the phonon density of states (base-centered tetragonal for HEC-V and base-centered tetragonal and some body-centered orthorhombic for HEC-W), i.e., these structures are dynamically unstable at 0 K, but may be stabilized at higher temperatures. Figure 1 shows the vibrational free energy $F_{\mathrm{vib}}$ and the vibrational entropy $S_{\mathrm{vib}}$ for both carbides and illustrates that the differences between including and not including the structures with imaginary frequencies is very small for HEC-V (less than 0.1 meV/atom and 0.01 $k_{\mathrm{B}}$/atom at 2000 K). For HEC-W, the error is larger: at 2000 K, $F_{\mathrm{vib}}$ is 7 meV/atom more positive and $S_{\mathrm{vib}}$ is 0.05 $k_{\mathrm{B}}$ smaller when structures with imaginary frequencies are included, which is partly due to the larger amount of imaginary modes. Near the transition temperatures (see Figure 4 in the main text), the differences in $F_{\mathrm{vib}}$ are below 1 meV/atom

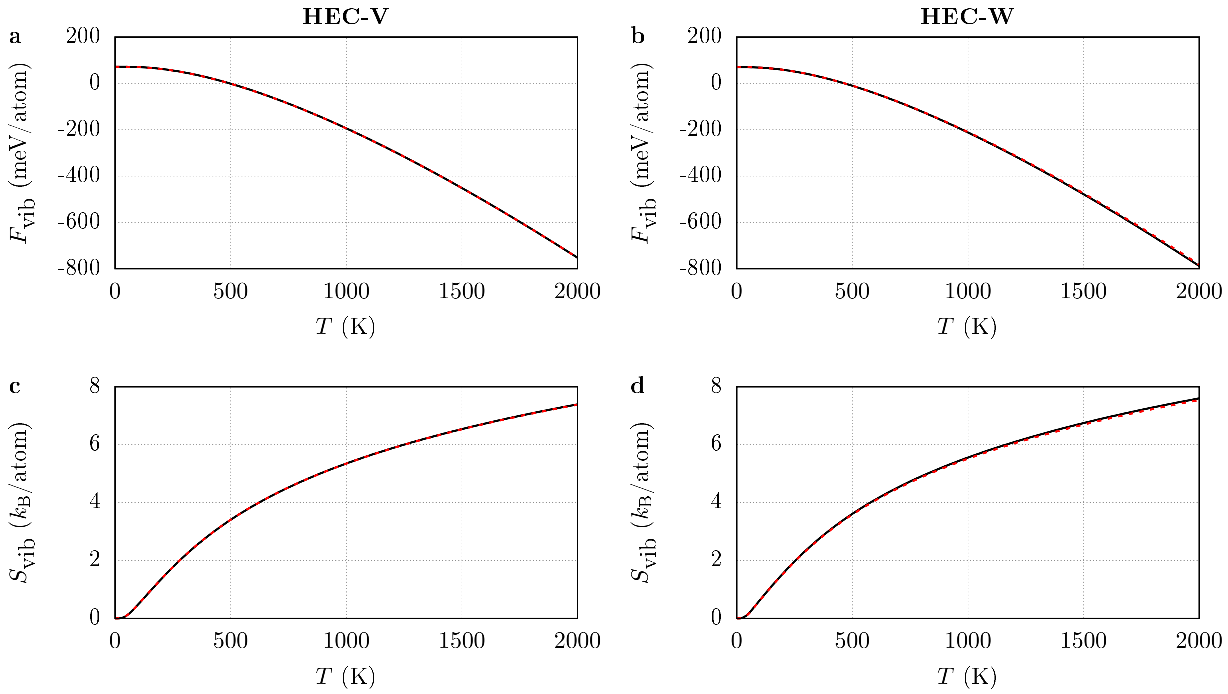


Supplementary Figure 1. **a-b** Vibrational free energy $F_{\mathrm{vib}}$ and **c-d** vibrational entropy $S_{\mathrm{vib}}$ for (HfNbTaTiV)C (HEC-V) and (HfNbTaTiW)C (HEC-W) including (red dashes) and not including (solid black) derivative structures with imaginary frequencies.


* stefano@duke.edu

so that removing these derivative structures from the ensemble average do not appreciably change the thermodynamics of the reactions.

## SUPPLEMENTARY NOTE 2: FREQUENCY SHIFTS IN HIGH-ENTROPY CARBIDES

Figure 2a-c show the phonon density of states (pDOS) of the high-entropy carbides and their rock-salt structure binary analogs at $T_{\mathrm{POCC}} = 2473$ K. The projected pDOS exhibit a shift in the metal pDOS compared to the binary carbides. Figure 2d shows the median frequency $\bar{\nu}$

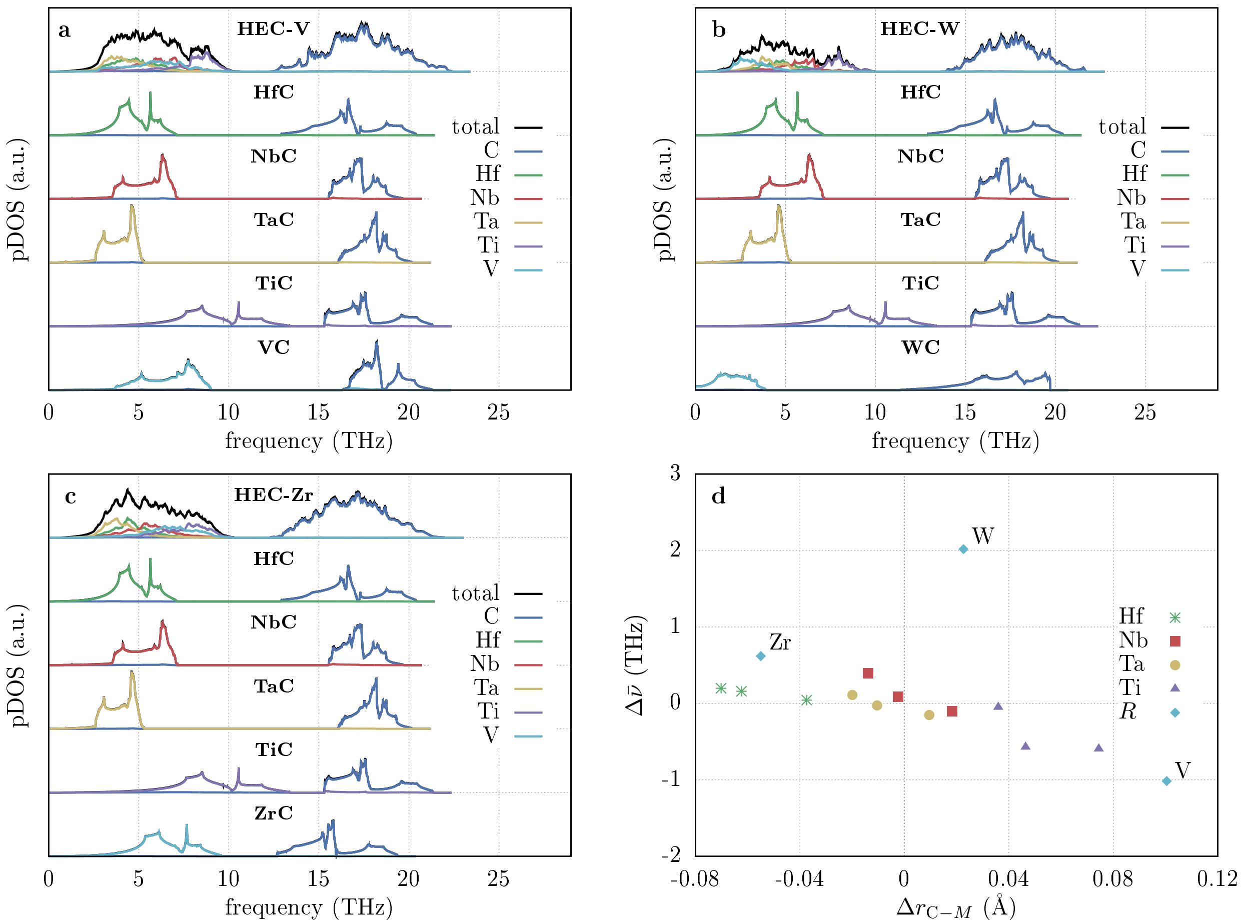


Supplementary Figure 2. **a-c** Total and projected phonon density of states for the for high-entropy carbides (HECs) (HfNbTaTi$R$) C (HEC-$R$) for $T_{\mathrm{POCC}} = 2473$ K and their their atomic projections. **d** Shift of the geometric center of the projected phonon density of states $\Delta\bar{\nu}$ of the metals in (HfNbTaTi$R$) C ($R = \mathrm{V, W, Zr}$) compared to rock-salt binary carbides as a function of the nearest-neighbor distance change $\Delta r_{\mathrm{C}-M}$.

of this shift as a function of nearest-neighbor distance changes ($\Delta r_{\mathrm{C}-M}$) with respect to the rock-salt binary carbides. The overall trend shows that the further the atoms are from their equilibrium positions the larger is the frequency shift. Metals that are further away from its nearest neighbors have its geometric center at lower frequencies and vice versa. These shifts differ from metal to metal, which may be the due to different bond strengths and/or to second-nearest neighbor (or higher order) interactions in the high-entropy material.

This trend explains the unexpected overlaps for vanadium and zirconium: V, which is 0.10 Å farther from its nearest neighbor in the HEC than in VC, shifts to lower frequencies by about 1 THz. Zr, being 0.06 Å closer to the surrounding carbides in the high-entropy material, shifts about 0.6 THz to higher frequencies. Deviating from this trend is tungsten with a significant upward frequency shift despite having an only slightly increased nearest-neighbor distance. This is primarily due to the large amount of imaginary modes in rock-salt WC that get removed in the high-entropy carbides.

## SUPPLEMENTARY NOTE 3: BINARY CARBIDE PHONON DISPERSIONS

Phonon dispersions were calculated for the binary carbide reactants HfC, NbC, TaC, TiC, VC, ZrC, and WC in the rock-salt structure (space group: $Fm\bar{3}m$, #225; Pearson symbol: cF8; AFLOW prototype[1–3]: AB_cF8_225_a_b[4]) with the finite difference method as implemented in the Automatic Phonon Library (APL) using a $5 \times 5 \times 5$ supercell (250 atoms). Phonon dispersions for WC were also calculated in the hexagonal ground state structure ($P\bar{6}m2$, #187; hP2; AB_hP2_187_d_a[5]) using a $5 \times 5 \times 4$ supercell (200 atoms).

Figure 3 shows the phonon dispersions of the binary carbides. Except for rock-salt WC, all compounds are dynamically stable. NbC, TaC, and VC exhibit phonon anomalies between the $\Gamma$ and $X$ and between $\Gamma$ and the $L$ point, which is consistent with findings from Li *et al.*[6]

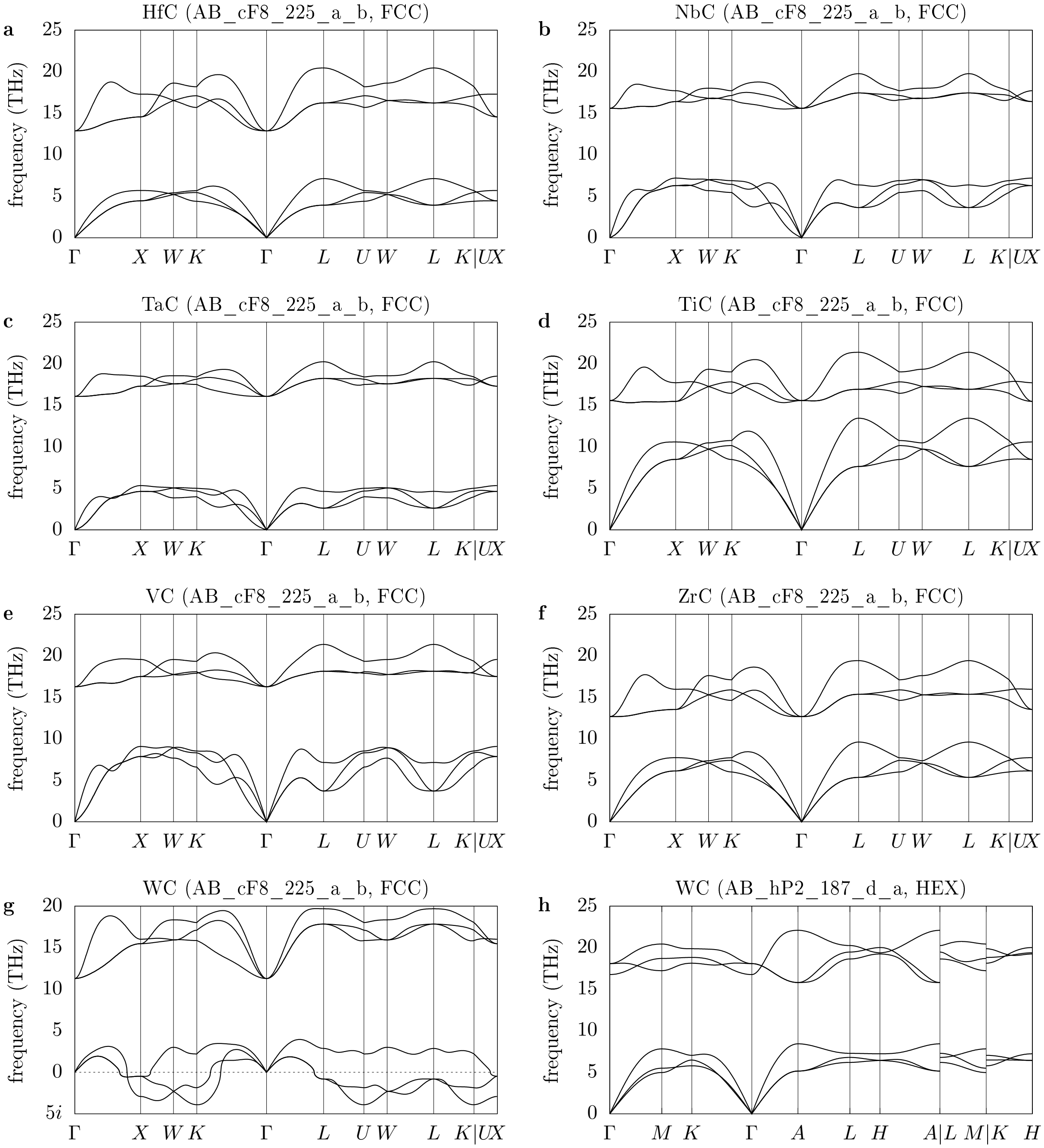


Supplementary Figure 3. Phonon dispersions of the binary carbides **a** HfC, **b** NbC, **c** TaC, **d** TiC, **e** VC, **f** ZrC, **g** WC in the rock-salt structure (AFLOW prototype AB_cF8_225_a_b[4]), and (h) WC in its experimental hexagonal structure (AFLOW prototype AB_hP2_187_d_a[5]).

## SUPPLEMENTARY REFERENCES

[1] M. J. Mehl, D. Hicks, C. Toher, O. Levy, R. M. Hanson, G. L. W. Hart, and S. Curtarolo, *The AFLOW Library of Crystallographic Prototypes: Part 1*, Comput. Mater. Sci. **136**, S1–S828 (2017), doi:10.1016/j.commatsci.2017.01.017.

[2] D. Hicks, M. J. Mehl, E. Gossett, C. Toher, O. Levy, R. M. Hanson, G. L. W. Hart, and S. Curtarolo, *The AFLOW Library of Crystallographic Prototypes: Part 2*, Comput. Mater. Sci. **161**, S1–S1011 (2019), doi:10.1016/j.commatsci.2018.10.043.

[3] D. Hicks, M. J. Mehl, M. Esters, C. Oses, O. Levy, G. L. W. Hart, C. Toher, and S. Curtarolo, *The AFLOW Library of Crystallographic Prototypes: Part 3* (2021), doi: 10.1016/j.commatsci.2021.110450.

[4] The AFLOW Library of Crystallographic Prototypes, *Rock Salt (NaCl, B1) Structure*, http://aflow.org/prototype-encyclopedia/AB_cF8_225_a_b.html (2019). (accessed April 30, 2019).

[5] The AFLOW Library of Crystallographic Prototypes, *Tungsten Carbide ($B_h$) Structure: AB_hP2_187_d_a*, http://aflow.org/prototype-encyclopedia/AB_hP2_187_d_a.html (2020). (accessed April 12, 2021).

[6] C. Li, N. K. Ravichandran, L. Lindsay, and D. Broido, *Fermi Surface Nesting and Phonon Frequency Gap Drive Anomalous Thermal Transport*, Phys. Rev. Lett. **121**, 175901 (2018), doi:10.1103/PhysRevLett.121.175901.
